# Developing a Novel Read-Across Concept for Ecotoxicological Risk Assessment of Phosphate Chemicals: A Case Study

**DOI:** 10.3390/toxics12010096

**Published:** 2024-01-22

**Authors:** Seokwon Lee, Seung-Yeop Ok, Hyo-Bang Moon, Sung-Chul Seo, Jin-Sung Ra

**Affiliations:** 1 Geum River Environment Research Center, National Institute of Environmental Research, Okcheon-gun 29027, Chungbuk, Republic of Korea; sukwon23@gmail.com; 2 Department of Environmental Fate and Modelling, Knoell Korea Ltd., Seoul 07327, Republic of Korea; osy7723@naver.com; 3 Department of Marine Sciences and Convergent Engineering, Hanyang University, Ansan 15588, Republic of Korea; hbmoon@hanyang.ac.kr; 4 Department of Nano, Chemical and Biological Engineering, College of Engineering, Seokyeong University, Seoul 02173, Republic of Korea; 5 Regulatory Chemical Analysis & Risk Assessment Center, Korea Institute of Industrial Technology (KITECH), Ansan 15588, Republic of Korea

**Keywords:** AChE inhibition, acute aquatic toxicity, octanol/water partition coefficient (Kow), phosphate chemicals, read-across assessment, species sensitivity factor

## Abstract

This study introduces a novel concept approach for a read-across assessment, considering species sensitivity differences among phosphate chemicals within structurally similar compound groups. Twenty-five organic chemicals, with a log Kow of 5 or less, were categorized into three functional groups based on acetylcholinesterase (AChE) inhibition as a specific mode of action (MOA). The short-term aquatic toxicity data (LC_50_) for fish, crustaceans, and insects were collected from the U.S. EPA Ecotoxicology (ECOTOX) Knowledgebase. A geometric mean calculation method was applied for multiple toxic endpoints. Performance metrics for the new read-across concept, including correlation coefficient, bias, precision, and accuracy, were calculated. Overall, a slightly higher overestimation (49.2%) than underestimation (48.4%) in toxicity predictions was observed in two case studies. In Case study I, a strong positive correlation (*r* = 0.93) between the predicted and known toxicity values of target chemicals was observed, while in Case study II, with limited information on species and their ecotoxicity, showed a moderate correlation (*r* = 0.75). Overall, the bias and precision for Case study I were 0.32 ± 0.01, while Case study II showed 0.65 ± 0.06; however, the relative bias (%) increased from 37.65% (Case study I) to 91.94% (Case study II). Bland–Altman plots highlight the mean differences of 1.33 (Case study I) and 1.24 (Case study II), respectively. The new read-across concept, focusing on AChE inhibition and structural similarity, demonstrated good reliability, applicability, and accuracy with minimal bias. Future studies are needed to evaluate various types of chemical substances, diverse modes of action, functional groups, toxic endpoints, and test species to ensure overall comprehensiveness and robustness in toxicity predictions.

## 1. Introduction

Over 84,000 chemicals are used in commerce around the globe, and new chemicals are produced every single day. Many chemicals have contributed to the development of various industries that improve our quality of life but, at the same time, increase the potential risk to human health and the environment, requiring rigorous approaches to ensure their safe use. The EU and U.S. introduced chemical regulations to assess toxicological effects and risks for the safer use of chemicals [1,2]. Risk assessment is a process designed to characterize potential risks and hazards of chemicals to human health and the environment upon exposure. The evaluation of chemical hazards is associated with information on physicochemical properties, structural similarity, and toxicity [3].

To assess the impact of chemical exposures on human and environmental health, animal testing data are generally necessary to gain insights into various biological systems [4]. In the context of the aquatic environment, toxicity data are used to derive the predicted no-effect concentration (PNEC), representing the concentration below which adverse effects are unlikely to occur during either long- or short-term exposure. However, due to the increasing number of chemicals, reliance on animal testing for the identification of chemical hazards and risks has become substantially limited. Ethical concerns, along with factors such as high cost, time constraints, and manpower, further contribute to these limitations [5]. Animal testing is also considered a poor predictor for humans [6]. Given these challenges and circumstances, non-animal testing techniques have been proposed in the field of environmental risk assessment and have been widely accepted all over the globe.

The EU, recognizing the need to minimize animal testing, considers it a last resort in chemical risk assessment and advocates for alternative methods. In recent decades, many countries, including South Korea, European nations, and the U.S., have also aimed to reduce their reliance on animal testing in chemical risk assessments. Read-across stands out as one of the alternative methods used to estimate various toxic endpoints for hazard assessment purposes. This approach predicts toxicological endpoints using the endpoint data of another chemical, provided that they are classified within the same analog chemical group. Chemicals are typically grouped based on structural similarity and other parameters related to the endpoint. The EU, U.S., and OECD have jointly published several technical guidance perspectives for the application of the read-across approach. In the context of aquatic toxicity, the structural similarity, chemical functional groups (FGs), mode of toxic actions (MOAs), and octanol–water partition coefficient (Kow) were considered [7,8,9].

In the traditional read-across approach, the aquatic toxicity value of a target chemical (with no available data) is simply predicted using the toxicity value of a source chemical within the same species when they belong to the same analog group. In a preliminary study, we assessed the performance of the traditional read-across approach for aromatic amines, a group considered for use in the read-across approach by the U.S. EPA for hazard assessments [10]. However, the differences between predicted and observed toxic values were up to 3.2 times greater in fish (*Pimephales promelas*) and 5.1 times greater in crustaceans (*Daphnia magna*), indicating potential limitations in terms of hazard assessment when using that approach.

According to the grouping method based on MOAs and FGs [11,12], we identified that these aromatic amines are mostly classified as a neutral organic group, their narcosis as a mode of toxic action. Given that narcosis is recognized as an unspecific mode of toxic action, there would be variations in the sensitivities among aquatic species, which are also affected by other factors [13]. Consequently, the traditional read-across approach, relying on the predicted toxicity values, may introduce potential uncertainty and lack reliability. To ensure high reliability and accuracy in toxicity predictions, using the read-across approach requires consideration of specific MOAs and species sensitivities. Furthermore, the correlation between MOAs and variations in aquatic species sensitivity has been extensively explored in ecological effect assessments, often employed for the estimation of species sensitivity distributions (SSDs) [14,15,16].

Therefore, in this study, we aimed to introduce a new concept approach for read-across assessments, considering the differences in species sensitivity to phosphate chemicals within structurally similar chemical compounds of the same category group and the specific mode of toxic action effects. We also evaluated the performance of a new read-across concept by calculating various statistical metrics, including the correlation coefficient, bias, relative bias, precision, and accuracy, and compared the differences between the log-transformed predictions and known toxicity values.

## 2. Materials and Methods

### 2.1. Selection of Chemicals in New Read-Across Concept Development

The subject chemicals were selected based on the parameters that are generally used for a chemical grouping of traditional read-across assessments of acute aquatic toxicity. The parameters were defined as the mode of toxic actions (MOAs), functional groups (FGs), and octanol–water partition coefficient (Kow) [7,8,9]. Among MOAs, we considered acetylcholinesterase (AChE) inhibition rather than unspecific MOAs (e.g., narcosis, etc.) because AChE inhibition exhibits a neuro route toxicity on particular biological molecules after chemical exposure [17,18]. An unspecific route of MOA may have limitations in its acute effects on aquatic species. Regarding the three FGs, ester (phosphate), oxime carbamate ester, and mono (or di) thiophosphate were considered, as they contribute to AChE inhibition through a neurotoxic mechanism [17,18]. If the chemicals possessed other functional groups unrelated to neurotoxic toxicity, they were not taken into consideration for chemical grouping. The MOAs and FGs of the chemicals were classified according to previous studies [11,12]. To determine the cut-off level of acute effect concentrations (LC_50_) in aquatic toxicity, log Kow, a factor of baseline toxicity, was employed. In general, aquatic toxicity tends to increase with the increased log Kow values reaching up to 5 to 6 [19,20]. In this log Kow range, the aquatic toxicity may be mainly affected by MOA together with the baseline toxicity. A total of twenty-five organic chemicals belonging to phosphate chemicals (*n* = 25) were selected based on the following specific criteria: a log Kow of less than 5, AChE inhibition, and being classified into three functional groups (Table 1).

### 2.2. Selection of Aquatic Species and Toxicity Data Collection

Target aquatic species were selected by considering the phylogenetic information related to AChE inhibition. According to phylogenetic proximity, aquatic species were classified into groups of similarity in terms of their nervous systems [21], and the classified species could be expected to show similar effects concerning the use of phosphate chemicals. Therefore, we considered fish, crustaceans, and insects as they also demonstrate AChE inhibition through specific neurotoxicity after chemical exposure [18,22,23]. We collected short-term (LC_50_) toxicity data for each of the three aquatic taxonomies through the U.S. EPA ECOTOX Knowledgebase [10]. The aquatic toxicity data were applied to calculate the geometric mean if more than three toxicity data were available for the same species in each taxonomy. In the process of collecting the toxicity data, we considered the water solubility limit, which explains the relationship between the dissolved concentration and uptake by aquatic species. Hence, we used an LC_50_ value less than the water solubility value.

### 2.3. Development of New Concept in Read-Across Assessment

In this study, we developed a novel concept relating to the read-across assessment method to address the limitations observed in traditional read-across approaches by considering species sensitivity factors, i.e., the differences in species sensitivity to the structurally similar chemical compounds that share a common mode of toxic action within the same category. We classified the target chemicals into three functional groups: ester (phosphate), carbamate, and thiophosphate, as those contributing to the neurotoxic mode of action (AChE inhibition) and toxicological mechanisms. A total of 25 organic chemicals with a log Kow of five or less were selected for chemical pairing, and then fish, crustaceans, insects, and amphibians were selected as the target species organisms for ecological risk assessment. Utilizing this new read-across approach, we predicted acute toxicity concentrations via pairwise matching of each chemical substance in a 1:1 manner.

### 2.4. Chemical Pairing for a New Read-Across Assessment

We paired a couple of chemicals to perform a new concept in the read-across assessment. In each paired couple, the two chemicals consisted of one source and another target chemical based on the log Kow difference. A chemical with a relatively higher log Kow than a source chemical was selected as a target chemical, while the paired source chemical was selected due to a lower log Kow than the target chemical. In this study, only the aquatic species with a high log Kow and increased ecotoxicity observed in the target chemical were included. Accordingly, we conducted a comparison concerning the differing levels of toxic concentrations for the common species among the chemicals to validate the variation in ecotoxicity corresponding to the increased log Kow values. In cases where toxicological information on the aquatic species was available (accessible), we identified a paired chemical group matching and then conducted the new read-across assessment concept. The toxicity data of each chemical were listed and matched with each other, whether the couple chemicals had the same species data or not. If the paired matching species data appeared to have higher toxic sensitivity to the source chemicals, it was not considered in our new read-across concept because the aquatic toxicity might be affected by a specific MOA and log Kow rather than other factors. The pairing of a couple of chemicals in the new read-across concept used one species from each taxonomy. According to this process, 25 target chemicals were applied to make a pairing couple of combinations to perform the new read-across concepts. Case study I limited the scenario, where the ecotoxicity data were only available for at least three of the four target aquatic species, and Case study II also limited the scenario, where the ecotoxicity data for at least two out of four aquatic species could be collected for use in the read-across assessment using the paired couple chemicals.

### 2.5. Introduction of a New Concept in the Read-Across Method with the Species Sensitivity Factor

**Figure 1:**
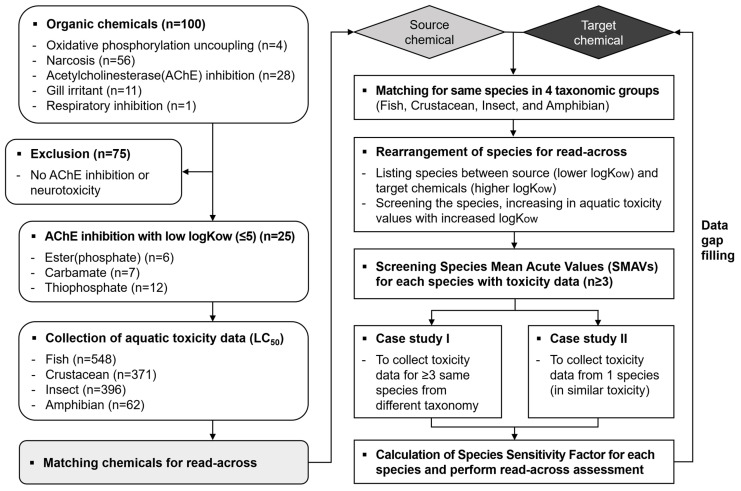
A flow chart diagram on the process of developing a new read-across assessment concept.

**Figure 2:**
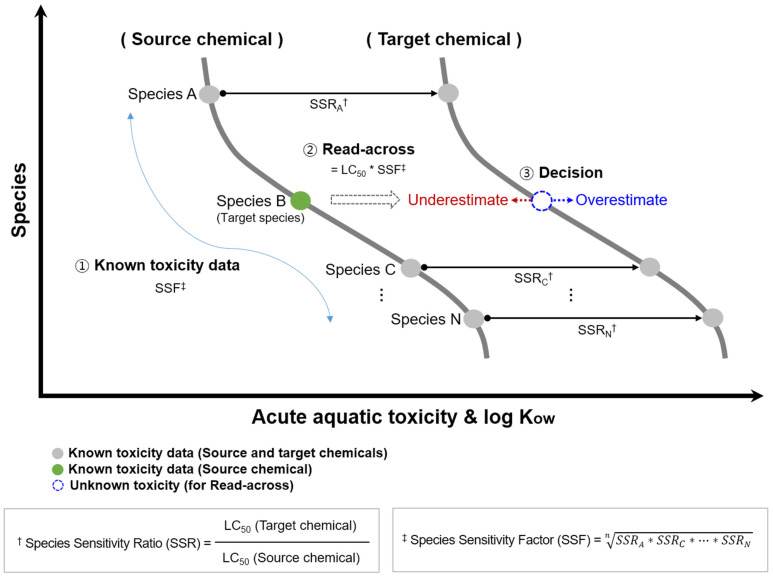
A conceptual framework designed for a newly developed read-across concept in assessing acute aquatic toxicity from source to target chemicals.

We introduced the species sensitivity factor (SSF) to the new read-across concept, which includes a couple of specific factors. SSF represents the geometric mean of species sensitivity ratios (SSRs), which are species-specific toxicity ratios for each paired matching chemical. The SSR of each species in the couple is derived by dividing the smaller LC_50_ value of the target chemical by the larger LC_50_ value of the source chemical. We developed two scenarios (i.e., Case studies I and II) for the new read-across concept according to the number of matched species between the source and the targeted coupled chemicals (Figure 1). We needed more than two species with known toxicity data in both the source and target chemicals and one species for the source chemicals, where we calculated SSR-1 and SSR-2 and further processed them for SSF, as in the Case study I approach. On the other hand, we needed one or more than one species (or simply uses a species showing the closest toxicity to the target species) with known toxicity data in the source and target chemicals and one species for the source chemical, as in the Case study II approach. The calculated SSF was applied to the toxicity value of the target species in the source chemical to predict the toxicity value of the target species in the target chemical. The results of the predicted toxicity value in the target chemical were assigned to either overestimation or underestimation by comparing the results to the observed toxicity data, which is assumed to be a data gap (Figure 2). Overestimation means that the predicted toxicity value shows the lowest toxicity value, i.e., greater toxic sensitivity than indicated in the observed toxicity data. Likewise, underestimation means that the predicted toxicity value is located between the observed toxicity data of the target chemical and the source chemical.

### 2.6. Calculation of Lack of Agreement

The lack of agreement (bias, relative bias, precision, and accuracy) was calculated to evaluate the performance of a new read-across concept approach to assess whether the predicted toxicity outcomes were over- or underestimated. Equations (1)–(3) are defined as follows [24]:

(1) $$Bias=\;\frac{1}{n}\;\sum_{j=1}^{n}\left(E_{j}-A\right)$$

(2) $$Relative\;bias=\;\left(e^{bias}-1\right)\times 100\%,\;where\;{e^{bias}\;is\;exp}^{\frac{\sum_{j=1}^{n}\left(E_{j}-A\right)}{n}}$$

(3) $$Precision=\;\frac{1}{n-1}\;\sum_{j=1}^{n}{\left[\left(E_{j}-A\right)-bias\right]}^{2}$$

where $E_{j}$ is the log-transformed predicted toxicity when using the new read-across concept for the $J^{th}$ sample. A is the log-transformed known toxicity value collected from the ECOTOX Knowledgebase. n is the number of the toxicity value present in the data set.

We also calculated the accuracy, defined as the mean absolute error (MAE), using Equation (4), which reveals the average distance between the predicted and known toxicity values. It is also known to be a less sensitive and more robust measure of accuracy [24]. We used the mean values of the predicted toxicity compared to the known toxicity values.

(4) $$Accuracy=\;\frac{1}{n}\;\sum_{j=1}^{n}\left|E_{j}-A\right|$$

Residuals, which are the differences between the log-transformed predictions and known toxicity values, were also calculated using Equation (5), as follows:

(5) $$Residual=\;E_{j}-A$$

We produced Bland–Altman plots showing the mean differences between the predicted and known toxicity values with 95% upper and lower limits of agreement. Finally, we calculated the Pearson’s correlation coefficients (*r*) between the log-transformed prediction and known toxicity values, and scatter plots with a fitted linear line with corresponding 95% confidence intervals (CIs) were also drawn for Case studies I and II.

### 2.7. Statistical Analysis

We statistically compared the predicted toxicity with the observed toxicity to examine the applicability of the new read-across concept approach. All read-across predicted and aquatic toxicity values were log-transformed after performing the Anderson–Darling normality test. Lack of agreement (bias, relative bias, precision, and accuracy), residuals, SSR, SSF, and Pearson’s correlation coefficients were calculated, and scatter plots with the fitted linear line and Bland–Altman plots were also drawn. All statistical analyses were performed using R statistical software version 4.2.2 (R Core Team, Vienna, Austria, 2023) with Rstudio version 2023.03.1+446 (Rstudio Inc., Boston, MA, USA), and a *p* value less than 0.05 was considered statistically significant.

## 3. Results

Table 1 shows the information on the toxicological mechanisms, physicochemical properties, and ecological toxicity classification for the 25 target chemicals related to AcheE inhibition included in this study. The three functional groups of chemical substances were classified as esters (phosphate), carbamates (subdivided into carbamate esters, phenyl, and oxime carbamate esters), and thiophosphates (mono or di). According to the classification criteria of Verhaar et al. (1992) [13], 19 chemicals were identified as Class 4 (specific-acting chemicals), while the rest of the chemicals were Class 5 (not considered). Furthermore, 22 chemicals (e.g., Dichlorvos, Mevinphos, etc.) were classified under aquatic acute hazard Category 1 (H400, H410) due to their chronic and acute toxic effects. One substance, Oxamyl (CAS no. 23135-22-0), was categorized as aquatic chronic hazard Category 2 (H411). Acephate (CAS no. 30560-19-1) and Dimethoate (CAS no. 60-51-5), on the other hand, were not classified as having aquatic hazard toxicity (Supplementary Table S1).

Table 2 shows the range of ecotoxicity values for each target chemical collected in this study. The concentration range of 24 h, 48 h, or 96 h LC_50_ of 72 species of fish such as *Oncorhynchus mykiss*, *Morone sapatilis*, and *Cyprinus carpio* was at least 6.93 × 10^−7^ to a maximum of 3.08 × 10^3^ mg/L, and the concentrations of 72 species of crustaceans, including *Daphnia magna*, *Palaemonetes pugio*, *Gammarus pulex*, etc., ranged from 7.28 × 10^−11^ to 2.35 × 10^3^ mg/L, respectively. The concentration range of 85 insect species, including *Aedes aegypti*, *Chauliodes* sp., and *Peltodytes* sp., was from 6.94 × 10^−7^ to 6.50 × 10^2^ mg/L, and the concentration range of 17 species of amphibians, such as *Ambystoma gracile*, *Microhylanata*, and *Rana boylii*, ranged from 9.40 × 10^−3^ to 8.82 × 10^3^ mg/L. The aquatic toxicity values of insect and amphibian species were not collected for some of the target chemicals, whereas all of the toxicity values on fish and crustaceans were collected from the U.S. EPA ECOTOX Knowledgebase for all 25 chemical substances.

In Table 3, the information concerning SSR across different species, known reference toxicity values collected from the U.S. EPA ECOTOX Knowledgebase, SSF, and predicted toxicities derived using the new read-across concept were summarized for seven target chemicals paired with source chemicals in the Case study I scenario. The predicted toxicity values showed a slightly higher percentage of overestimation (49.2%) than underestimation (48.4%), and in three substances, Carbaryl, Malathion, and Chlorpyrifos, the outcomes in over- and underestimations in terms of species were the same: thus, the decision concerning either over- or underestimation was not made. In Table 4, the predicted toxicity values and over- or underestimation decisions are shown for 10 target chemicals using the new read-across approach. In the Case study II scenario, however, the predicted toxicity values showed a higher overestimation percentage (52%) than underestimation (48%). Therefore, the predicted toxicity values using the new read-across concept presented a tendency toward overestimation when comparing the known toxicity values in both scenarios (Case studies I and II).

**Figure 3:**
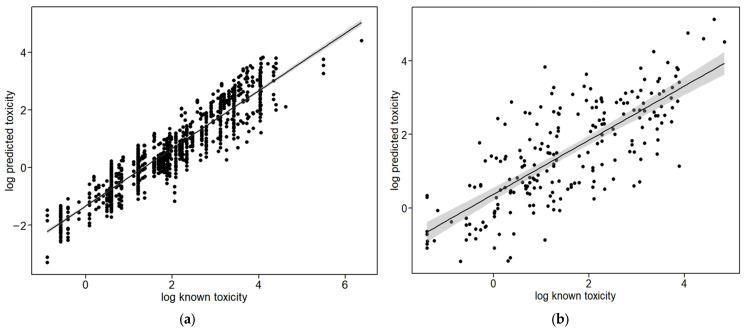
Scatter plots for the log-transformed predicted and known toxicity values in the fitted line with 95% confidence intervals (CIs) (grey-colored): (**a**) Case study I (n = 1095, *r* = 0.93); (**b**) Case study II (n = 218, *r* = 0.75).

In Table 5, the results yielded from calculating the overall bias, relative bias, precision, and accuracy are shown. The overall bias and precision were 0.32 ± 0.01 for Case study I and 0.65 ± 0.06 for Case study II, respectively. Relative bias (%) was 37.65% for Case study I and 91.94% for Case study II, indicating that both Case studies I and II scenarios showed overestimation. The overall accuracy was 0.01 for Case study I and 0.05 for Case study II, indicating that both of the case studies had high accuracy. In Case study I, the bias and precision for a functional group of thiophosphates were as high as 0.01 ± 0.01, with an accuracy of 0.004. Figure 3 also shows scatter plots demonstrating the linear relationships between the predicted and known toxicity values in the target chemicals derived using a new read-across concept approach. The Pearson correlation coefficient was *r* = 0.93 for Case study I, indicating a strong positive correlation (Figure 3a), and a moderate correlation (*r* = 0.75), which is relatively lower than that for the Case study I scenario, was observed in Case study II (Figure 3b).

**Figure 4:**
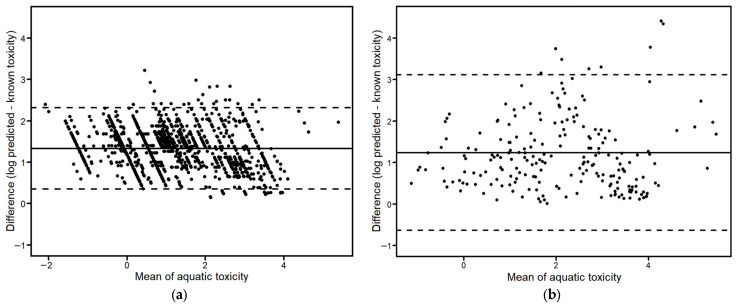
Bland–Altman plots for differences between the predicted and known toxicity data. The mean differences with 95% upper and lower limits of agreement are marked with the dashed lines: (**a**) Case study I (n = 1095): Mean = 1.33, +1.96 SD = 2.32, −1.96 SD = 0.35; (**b**) Case study II (n = 218): Mean = 1.24, +1.96 SD = 3.11, −1.96 SD = −0.64.

In Figure 4, the Bland–Altman plots were drawn to demonstrate the mean differences between the predicted and known toxicity values. For the Case study I scenario, the mean difference was 1.33, and most of the predicted values were within a 95% upper and lower limits range (+1.96 SD 2.32~−1.96 SD 0.35); however, some appeared to be outside of the range. On the other hand, the mean difference was 1.24 for the Case study II scenario, which was lower than the Case study I. Nonetheless, the 95% upper and lower limits ranged from +1.96 SD 3.11 to −1.96 SD −0.64, which is relatively wider than that of Case study I. Only eight values exceeded the 95% upper limit value in all of the datasets.

## 4. Discussion

In this present study, we developed a novel read-across concept approach to assess predicted toxicity by considering differences in species-specific sensitivity, known as species sensitivity factors. We used a dataset of 25 organic chemicals with AChE inhibition (MOA). To account for interspecies variation, we collected known reference toxicity values for fish, crustaceans, and insects from the U.S. EPA ECOTOX Knowledgebase. In the first scenario (Case study I), we had a minimum of one or more toxicity data values available per species (i.e., at least three or more species’ toxicity information can be collected and used in common), and the predicted outcomes revealed a strong positive correlation (*r* = 0.93) with known toxicity values. In the second scenario, however, where toxicity-related information was limited for some species (i.e., only available for some species), we observed a moderate correlation (*r* = 0.75), indicating that the first scenario (Case study I) had higher predictability and reliability.

Generally, there are four types of read-across assessment approaches, each contingent on data availability: one-to-one, one-to-many, many-to-one, and many-to-many read-across. For this study, we adopted a one-to-one approach, anticipating the toxicity of target chemicals based on the toxicity of the source chemicals. As outlined in this study, we endeavored to enhance the reliability of our findings by incorporating other aquatic species with known toxicity values that exhibit biologically or genetically similar toxic mechanisms. In Case study I, we employed two or more different species, while in Case study II, we utilized only one species. The key distinction between these two approaches lies in the number of species with known toxicity values. We posit that a higher number of such species improves prediction accuracy, aligning with our assumptions. It is important to note the variability in toxicity values of biological origin, which can differ significantly across laboratories or experiments. Occasionally, such disparities are so pronounced that scientists may omit certain values as outliers. While this issue falls outside the scope of our study, as experimental errors are inherently present in the existing toxicity values, it is worth recognizing that the pattern of toxicity values may conform to a normal distribution with a larger volume of increased datasets. In this regard, our study does not set boundaries based on the number of preexisting datasets in aquatic species, but the inclusion of a greater variety of species may contribute to the overall reliability of our findings.

Furthermore, the predicted outcomes from the two scenarios generally demonstrated a tendency toward overestimation. In Case study I, the overestimated values of acute aquatic toxicity were approximately 40% higher than the known reference values. However, in Case study II, this overestimation was much higher, at approximately 90%. Therefore, the newly developed read-across approach demonstrates good accuracy, precision, and high reliability in predicting acute aquatic toxicity for organic chemicals at a screening level. In fact, the prediction of aquatic toxicity using a new read-across concept approach was based on the hypothesis that toxicity is primarily caused by structural similarity, the mode of toxic action, toxicological mechanisms, and log Kow, among other parameters. In this study, the mode of toxic action for organic substances was based on one of the neurotoxic mechanisms, AChE inhibition. Colovic et al. (2013) [18] reported that irreversible AChE inhibition is associated with specific chemical functional groups, including ester (phosphate), carbamates, and mono- or di-thiophosphates, i.e., organophosphorus compounds.

Nagai and Taya [16] observed a relationship between the average sensitivity of algae and EC_50_ values for herbicides. They reported that when the specific MOA information within the same category was taken into consideration, the accuracy of predicting sensitivity variation, known as species sensitivity distribution (SSD), significantly improved. In this present study, we similarly considered the differences in toxicity sensitivity among various aquatic species for organic substances exhibiting AChE inhibition as their common mode of action. Our study indicates that the accuracy of the predicted results was high, which is consistent with that found in the previous studies. In addition, a study conducted by Lambert et al. [25] performed acute toxicity concentration (LC_50_) predictions for 617 organic substances, considering the log Kow values. The study found that the accuracy of the toxicity prediction results was significantly higher in the specific MOA-based QSAR models compared to a wide range of general MOA groupings. Therefore, the authors of this study have provided sufficient evidence that log Kow, one of the fundamental physicochemical characteristics, is significantly correlated with the acute toxicity of organic matter across diverse ranges of MOA categories, which is similar to two previous studies [25,26]. Belanger et al. [27] also noted a robust linear correlation between the acute toxicity of short- and long-chain alcohols to fish and log Kow (hydrophobicity).

In line with previous studies, our current study identified that the log Kow values of the organic substances selected for the newly developed read-across assessment fell within the range of −0.85 to 4.96 rather than exceeding a cutoff range. Furthermore, we also observed a correlation whereby the average toxicity value of each species was associated with an increase in the log Kow values (resulting in lower toxicity values) for organic substances. Suter and Lizarraga [28] provided evidence for read-across assessments, which were classified into four broad categories: structural features of chemicals, physicochemical properties, toxicokinetics, and toxicodynamics. In particular, they emphasized that source–target chemical analogs should be evaluated for similarity across various parameters, including taxonomy, toxic endpoint, exposure route, structural features, toxicokinetic or toxicodynamic pathways and processes, structure–activity relationships, mechanisms of toxicity, etc. Therefore, a Weight of Evidence (WoE) assessment for read-across assessments should be conducted to examine whether there is relevance, strength, or reliability among these parameters, ultimately enhancing the credibility and validity of the entire assessment process and results.

In the present study, we quantitatively predicted the LC_50_ concentrations of target substances by pairing source and target chemicals with one-on-one (1:1) couple matching based on similar functional groups with respect to structurally similar characteristics, toxic mode of action (AChE inhibition), functional groups, octanol–water partition coefficient (Kow), toxicity effects, and endpoints (i.e., acute aquatic toxicity). Therefore, prior to our new read-across concept applied to aquatic toxicity prediction and assessment, our hypothesis satisfied the results reported in several previous studies, thus leading to a high level of reliability in terms of the predicted results with high confidence. Therefore, it can be concluded that a new read-across concept could be relevant to predicting aquatic toxicity with high reliability, accuracy, and less bias. Several previous studies have also reported predictions of acute toxicity for various organic substances using the refined read-across methods, which are similar to the read-across concept approach employed in this study.

For instance, Kühne et al. [29] utilized a read-across approach applying Kow- and LSER-based methods to predict the acute toxicity concentration (48 h-LC_50_) for water fleas, and they observed a strong correlation between their predictions and experimental data, indicating that their predictions were within the applicability domain. Chavan et al. [30] used a k-nearest neighbor (k-NN) classification method that they developed to determine the most optimal k value. Then, they classified the substances into two classes (e.g., highly toxic or non-harmful) based on their LD_50_ values and predicted repeated dose toxicity (LOEL) values. These model predictions demonstrated good applicability, with approximately 74% within an order of magnitude of the experimental LOEL values. In another study, Paul et al. [31] collected soil pEC50 values of *F. candida* for various classes of organic compounds from the U.S. ECOTOX Knowledgebase and used diverse prediction models to estimate the toxicity values. In particular, when using the chemical read-across method to predict unknown toxicity values of similar substances based on known toxicity values of substances within the same category, the results showed much better-predicted values in terms of validation metrics compared to the intelligent consensus model (i.e., the partial least square PLS model). This read-across approach was reported to be a practical tool for predicting toxicity, using small datasets, and was found to be simple, accurate, and reliable.

Furthermore, Lizarraga et al. [32] recently introduced a modified, revised read-across framework based on key principles, evaluation experience, and new approach methodologies (NAMs) data. The revised read-across framework particularly emphasizes evaluating biological similarity as a means of identifying groups of similar substances. The sequence of read-across assessment has five steps, and in the third step, the structural similarity, toxicokinetic/toxicodynamic properties, similar toxic pathways and effects, and candidate similar substances are compiled or grouped within the same category (i.e., similar functional groups). Therefore, we anticipate that the results obtained by applying our new read-across concept approach, appropriately incorporating the key principles and steps in our new read-across assessment process and methodology, will provide scientifically valid evidence to predict the acute aquatic toxicity of organic substances focusing on AChE inhibition.

However, there are some limitations to this study. First, the sample size of the selected organic chemicals for the read-across assessment was relatively small (*n* < 30) since we established three hypotheses for the study goal: a range of log Kow less than 5, a specific MOA of AChE inhibition, and a category for chemical functional groups. Secondly, some of the aquatic species in each taxonomy show higher toxicity to the source chemicals that have a lower log Kow value than the target chemicals. This suggests that other species may have different sensitivities to chemicals, even though the same MOA contributes significantly to species sensitivity. In fact, the available toxicity information was collected and evaluated considering the physicochemical characteristics of specific organic substances and their toxic mode of action only. Therefore, it is difficult to generalize and apply this new read-across concept to all other chemical substances, various toxic modes of action, different toxic endpoints, and circumstances. The other types of MOAs for aquatic species should also be applied to assess aquatic toxicity using the new read-across approach. Apart from AChE inhibition, other types of MOAs with specific endpoints and toxic effects have also been widely studied in previous studies concerning respiratory blocking [17,33], irritants [17], central nervous system seizing [34], and inhibiting oxidative phosphorylation [35,36]. Most studies provided sufficient evidence of the relationship between MOAs and variations in species sensitivity based on chemical functional groups [14,15,16]. Thus, further studies are necessary to consider other well-known MOAs to apply this new concept to diverse functional groups and different types of chemical substances and validate this new read-across concept approach using a larger number of predicted toxicity values in various situations in the future.

Despite these limitations, this study has several strengths. First, we fully satisfied the assessment process, steps, and key principles of the newly revised read-across framework. We collected all the available data for predicting the aquatic toxicity of target chemicals by using a large-scale dataset from the globally recognized and well-known U.S. EPA ECOTOX Knowledgebase. In this study, we performed one-on-one (1:1) couple pairing of the source and target chemicals, with the collection of large groups of known acute toxicity (LC_50_) values for various test species widely used in the OECD test guidelines, such as *Danio rerio*, *Oryzias latipes*, etc., for fish, *Daphnia magna*, etc., for crustaceans, and *Chironomus* sp., etc., for insects in Case study I (n = 1095) and II (n = 218), respectively. Therefore, our new read-across concept introduced highly reliable performances in toxicity prediction by applying a sufficient large-scale dataset of aquatic toxicity, which is essential for the statistical evaluation of accuracy, bias, precision, and reliability in this study. However, it is unable to quantitatively compare our study results with previous studies due to the unavailability of raw data. If possible, a new pooled analysis using all combined data and related information acquired from the previous and current studies should be performed to increase the credibility, accuracy, and validity of the new read-across concept.

Moreover, the U.S. EPA ECOTOX Knowledgebase, which we used to collect toxicity information, offers a strong advantage in terms of the comprehensive and transparent curation of detailed aquatic toxicity datasets from the latest study results, following a rigorous process of literature search and systematic review. It also provides extensive updates and encompasses a wide range of documents, including all endpoints and other related toxic effects, rendering it a reliable and comprehensive source of aquatic toxicity data. This database is made readily accessible to all stakeholders, including government officials, academic researchers, and industrial experts, by ensuring ease of use, free of charge, accessibility, and a high level of data quality.

In a recent review, Olker et al. [37] reported that the ECOTOX database (Version 5) included the ecotoxicity data for over 12,000 chemicals and 13,000 species with over one million testing results in the last decade and a higher volume of data updates. All toxicity data underwent internal review and were subjected to quality assurance (QA) processes. More importantly, this database serves as a curated source of toxicity data that offers high reliability and applicability for various chemical risk assessments and related validation studies. It can also be interoperable with other available toxicity databases, continuously evolving into a state-of-the-art source of information and practices around the globe.

Most importantly, this study considered inter-species sensitivity using the aquatic toxicity information for various organisms collected from the recently published references and toxicity databases. We also proposed a new concept approach of read-across assessment that enables the simple and rational prediction of acute aquatic toxicity concentrations of specific target substances at a screening level. Using this method, further studies can be conducted to predict, evaluate, and be applied to diverse chemical substances, different toxic mechanisms (MOAs), and other toxic endpoints and effects, thus enabling further cross-validations of this method (prediction model) of the new read-across assessment that we have introduced in the present study.

In particular, further studies should focus on using AI-based toxicity prediction techniques to predict aquatic toxicity values for various toxic endpoints of organic substances in various MOA groups, as indicated by the recent trends in the published literature. A comprehensive review conducted by Jeong and Choi [38], covering research papers on toxicity prediction and risk assessment from 1990 to 2020, highlighted the increasing use of machine and deep learning models. These advanced models, including algorithms such as random forest, support vector machines, and deep neural networks, can be employed to predict various toxicity endpoints in aquatic environments. Given that AI-based prediction models can handle large volumes of toxicity data, it is anticipated that they will play an important role in read-across approaches and validation as larger datasets can be collected in the future.

Certainly, while models developed using artificial neural networks (ANNs) may not fully replace experimental toxicity data, they are expected to serve as valuable screening tools in situations where experimental data are lacking or insufficient [39]. In addition, similarity-based machine learning models successfully predicted acute toxicity concentrations for various organic chemicals in two species, *D. magna* and *O. latipes*, demonstrating a high correlation with experimental data [40]. The application of the newly developed read-across structure–activity relationship (RASAR) model demonstrated more accurate, consistent, and reproducible predictions of aquatic acute toxicity values, with the species identified as the most crucial input feature [41].

Although it is nearly impossible to fully predict exposure scenarios for chemical mixtures or complexes in real-world environments, machine learning-based models would be applicable. Recently, a modeling study conducted by Schür et al. using the ADORE datasets extracted from the U.S. EPA ECOTOX database utilized machine learning techniques to predict acute ecotoxicity. The detailed modeling processes, approaches, and predicted outcomes, including model performance and best practices, were also described [42]. Therefore, it is highly expected that further studies on machine learning-based techniques for ecotoxicity predictions will provide evidence of model improvement and cross-validation with less uncertainty in the future.

## 5. Conclusions

In this study, the new read-across assessment concept demonstrated a strong correlation with known reference toxicity, indicating high reliability and accuracy in predicting aquatic toxicity for target substances with minimal bias. We focused on AChE inhibition as a specific mode of action (MOA) and structural similarity (functional groups) across three aquatic species, including fish, crustaceans, and amphibians. Our concept followed a modified read-across framework, considering important elements, such as source–target chemical paring, species sensitivity factors, species sensitivity ratio (SSR), and the species sensitivity factor (SSF). Future studies should emphasize different substances, diverse toxic action modes, various functional group categories, additional toxic endpoints, and effects on various test species. Further validation of the read-across concept approach is crucial, and comparative studies using machine learning-based models should be conducted to ensure overall comprehensiveness and robustness in ecotoxicity predictions.

## Figures and Tables

**Table 1:** Physicochemical properties and classification of aquatic toxicity for 25 target chemical substances included in this study. [All pictures of chemical structures were downloaded from the official website of PubChem (https://pubchem.ncbi.nlm.nih.gov/, accessed on 1 September 2023)].

No	Mode of Action (MOA)	Chemical Functional Group	Subgroup	Chemical Name	CAS no.	Molecular Weight (g/mol)	Log Kow	Water Solubility (mg/L)	Activity Class
1	AChE inhibition	Esters (phosphate)	-	Acephate	30560-19-1	183.16	−0.85	818.00	Class 5
2				Dichlorvos	62-73-7	220.97	1.43	8.00	Class 5
3				Methamidophos	10265-92-6	141.13	−0.80	1000.00	Class 5
4				Mevinphos	7786-34-7	224.15	0.13	600.00	Class 5
5				Nemacur	22224-92-6	303.36	3.23	0.33	Class 5
6				Profenofos	41198-08-7	373.63	4.68	0.03	Class 5
7		Carbamate	Carbamate Esters, Phenyl	Aminocarb	2032-59-9	208.26	1.90	0.92	Class 4
8				Carbaryl	63-25-2	201.22	2.36	0.11	Class 4
9				Carbofuran	1563-66-2	221.25	2.32	0.32	Class 4
10				Propoxur	114-26-1	209.24	1.52	1.86	Class 4
11			Oxime Carbamate Ester	Aldicarb	116-06-3	190.26	1.13	6.03	Class 4
12				Methomyl	16752-77-5	162.21	0.60	58.00	Class 4
13				Oxamyl	23135-22-0	219.26	−0.47	280.00	Class 4
14		Thiophosphate	Mono	Chlorpyrifos	2921-88-2	350.59	4.96	0.001	Class 4
15				Diazinon	333-41-5	304.35	3.81	0.04	Class 4
16				EPN	2104-64-5	323.31	4.78	0.003	Class 4
17				Fensulfothion	115-90-2	308.35	2.23	2.00	Class 4
18				Fenthion	55-38-9	278.32	4.09	0.01	Class 4
19				Methyl parathion	298-00-0	263.21	2.86	0.04	Class 4
20			Di	Azinphos-methyl	86-50-0	317.32	2.75	0.02	Class 4
21				Dimethoate	60-51-5	229.25	0.78	23.30	Class 4
22				Disulfoton	298-04-4	274.39	4.02	0.02	Class 4
23				Dyfonate	944-22-9	246.32	3.94	0.02	Class 4
24				Malathion	121-75-5	330.35	2.36	0.14	Class 4
25				Terbufos	13071-79-9	288.42	4.48	0.01	Class 4

**Table 2:** Acute aquatic toxicity data were collected from the ECOTOXicology Knowledgebase (ECOTOX, Version 5, U.S. EPA, 2022) for 25 target chemicals. * LC_50_ (mg/L): All of the available toxicity values (either 24 h, 48 h, or 96 h-LC_50_) were selected and collected from the ECOTOX database.

No	Chemical Functional Group	Chemical Name	Fish		Crustacean		Insect		Amphibian	
			n	LC_50_ * (mg/L)	n	LC_50_ * (mg/L)	n	LC_50_ * (mg/L)	n	LC_50_ * (mg/L)
1	Esters (phosphate)	Acephate	12	1.46 × 10^−3^~3.08 × 10^3^	7	3.50 × 10^−1^~2.35 × 10^3^	9	1.14 × 10^−1^~6.50 × 10^2^	2	6.43 × 10^3^~8.82 × 10^3^
2		Dichlorvos	38	6.41 × 10^−3^~1.67 × 10^2^	25	1.30 × 10^−4^~1.29 × 10^2^	24	1.00 × 10^−4^~2.38 × 10^2^	5	7.80 × 10^−1^~7.89 × 10
3		Methamidophos	8	5.36 × 10 ~1.12 × 10^2^	4	1.61 × 10^−6^~1.46	-	-	1	2.72 × 10
4		Mevinphos	12	2.23 × 10^−2^~4.00 × 10	9	9.50 × 10^−4^~1.30 × 10^−1^	1	4.95 × 10^−3^	-	-
5		Nemacur	3	1.75 × 10^−2^~1.40 × 10^−1^	6	2.65 × 10^−3^~1.50 × 10^−1^	-	-	-	-
6		Profenofos	17	2.55 × 10^−3^~2.02	11	4.10 × 10^−5^~7.71	6	1.18 × 10^−2^~3.70	1	5.80 × 10^−1^
7	Carbamate	Aminocarb	12	5.24 × 10^−1^ ~1.00 × 10^2^	8	1.20 × 10^−2^~3.27 × 10	7	2.25 × 10^−2^~2.86	-	-
8		Carbaryl	60	6.93 × 10^−7^~1.08 × 10^2^	41	3.75 × 10^−3^~9.65	42	6.94 × 10^−7^~4.79	17	1.64~5.53 × 10
9		Carbofuran	28	4.23 × 10^−2^~7.90	15	3.32 × 10^−4^~2.70	8	1.19 × 10^−4^~1.59	3	1.12 × 10~1.13 × 10^2^
10		Propoxur	12	1.30~4.25 × 10	7	1.50 × 10^−2^~1.43	22	1.80 × 10^−2^~8.00	-	-
11		Aldicarb	9	6.56 × 10^−2^~4.50 × 10	16	1.20 × 10^−2^~1.73 × 10	5	2.00 × 10^−2^~2.80 × 10^−1^	-	-
12		Methomyl	14	3.40 × 10^−1^~5.25	19	6.40 × 10^−3^~7.20	4	6.43 × 10^−2^~8.79 × 10^−1^	3	5.55 × 10~6.16 × 10^2^
13		Oxamyl	6	2.60~2.75 × 10	4	2.20 × 10^−1^~2.30 × 10	-	-	-	-
14	Thiophosphate	Chlorpyrifos	40	5.80 × 10^−4^~5.98 × 10^−1^	47	7.28 × 10^−1^~ 4.57 × 10^−1^	85	5.00 × 10^−5^~3.80 × 10^−1^	6	1.90 × 10^−2^~5.62 × 10^−1^
15		Diazinon	53	7.00 × 10^−5^~4.00 × 10	20	3.87 × 10^−4^~1.25	23	5.67 × 10^−4^~2.20 × 10^−1^	5	3.43~1.48 × 10
16		EPN	11	1.81 × 10^−2^~4.20 × 10^−1^	6	2.90 × 10^−4^~4.38 × 10^−2^	2	1.10 × 10^−3^~7.40 × 10^−3^	-	-
17		Fensulfothion	3	7.20 × 10^−2^~4.31 × 10	2	1.00 × 10^−2^~5.07 × 10^−2^	-	-	-	-
18		Fenthion	21	4.53 × 10^−1^~3.10	16	1.52 × 10^−4^~1.80	57	5.00 × 10^−4^~1.10	1	9.40 × 10^−4^
19		Methyl parathion	42	5.00 × 10^−3^~1.90 × 10	28	2.05 × 10^−4^~4.00	12	5.40 × 10^−4^~2.74 × 10^−1^	4	3.70~9.59
20		Azinphos-methyl	26	3.60 × 10^−4^~4.06	10	1.61 × 10^−4^~1.38 × 10	6	3.70 × 10^−4^~8.85 × 10^−2^	-	-
21		Dimethoate	26	1.30 × 10^−1^~4.20 × 10	14	2.00 × 10^−3^~1.50 × 10	13	1.29 × 10^−3^~9.60	5	7.82 × 10^−3^~3.75 × 10
22		Disulfoton	10	3.70 × 10^−2^~7.20	3	3.90 × 10^−3^~1.00 × 10^−1^	1	5.00 × 10^−3^	-	-
23		Dyfonate	5	1.60 × 10^−2^~1.09	2	4.90 × 10^−4^~2.70 × 10^−3^	2	3.90 × 10^−2^~5.40 × 10^−2^	-	-
24		Malathion	72	1.95 × 10^−2^~1.70 × 10	46	9.70 × 10^−5^~8.15 × 10	67	1.00 × 10^−3^~2.69 × 10	9	5.90 × 10^−4^~3.32 × 10
25		Terbufos	8	2.90 × 10^−3^ ~1.31 × 10^−1^	5	2.97 × 10^−4^~1.00 × 10^−2^	-	-	-	-

**Table 3:** (Case study I) The example of results of the predicted values of acute aquatic toxicity (in read-across) for target chemicals using known toxicity data of source chemicals. * n: Number of species in fish/crustacean/insect, ** ND: Not determined for either over- or underestimation of predicted toxicity.

No	Chemical Functional Group	n *	Species	Source Chemical	Target Chemical	Known Toxicity of Target Chemical (ug/L)	Predicted Toxicity of Target Chemical (Read-Across) (ug/L)		Known/Predicted Toxicity Decision Ratio of Example			Known/Predicted Toxicity Decision Ratio of Each Species		
							Min	Max	Overestimation (n)	Underestimation (n)	Decision	Decision of Over estimation (n)	Decision of Under estimation (n)	Decision of ND ** (n)
1	Esters (phosphate)	1/3/1	Fish (e.g., *Gambusia affinis*)	Acephate	Profenofos	3.48 × 10^2^	1.82 × 10^3^	5.79 × 10^3^	0	3	Under	0	1	0
			Crustacean (e.g., *Americamysis bahia*)			2.37	7.85 × 10	-	0	1	Under	0	3	0
			Insect (e.g., *Culex quinquefasciatus*)			1.18 × 10	6.84 × 10^−2^	2.18 × 10^−1^	3	0	Over	1	0	0
2	Esters (phosphate)	2/4/3	Fish (e.g., *Gambusia affinis*)	Dichlorvos	Profenofos	3.48 × 10^2^	7.57 × 10	2.91 × 10^3^	5	7	Under	0	2	0
			Crustacean (e.g., *Americamysis bahia*)			1.71	8.93 × 10^−2^	5.72	2	4	Under	2	2	0
			Insect (e.g., *Culex pipiens *ssp.* Pallens*)			7.45 × 10	1.85	2.56 × 10	8	0	Over	2	1	0
3	Carbamate	4/1/1	Fish (e.g., *Oncorhynchus mykiss*)	Propoxur	Carbaryl	1.21 × 10^3^	6.50 × 10^3^	-	0	1	Under	2	2	0
			Crustacean (e.g., *Daphnia magna*)			2.07 × 10^2^	8.47 × 10	2.17 × 10^2^	3	1	Over	1	0	0
			Insect (e.g., *Aedes aegypti*)			7.08 × 10^2^	3.21 × 10^2^	8.22 × 10^2^	2	2	ND **	0	0	1
4	Carbamate	4/1/1	Fish (e.g., *Salvelinus fontinalis*)	Aminocarb	Carbaryl	1.88 × 10^3^	7.09 × 10^2^	-	1	0	Over	2	2	0
			Crustacean (e.g., *Gammarus pseudolimnaeus*)			1.53 × 10	6.39 × 10	1.15 × 10^2^	0	4	Under	0	1	0
			Insect (e.g., *Pteronarcella badia*)			1.53 × 10	3.29	5.88	4	0	Over	1	0	0
5	Thiophosphate	3/2/1	Fish (e.g., *Cyprinus carpio*)	Dimethoate	Malathion	2.65 × 10^3^	5.80 × 10	7.03 × 10^2^	2	0	Over	2	0	1
			Crustacean (e.g., *Daphnia magna*)			7.28	3.51 × 10	2.12 × 10^2^	0	3	Under	1	1	0
			Insect (e.g., *Aedes aegypti*)			1.06 × 10^2^	3.56 × 10	2.60 × 10^3^	1	5	Under	0	1	0
6	Thiophosphate	4/1/1	Fish (e.g., *Oreochromis niloticus*)	Dimethoate	Chlorpyrifos	1.25 × 10^2^	3.63	-	1	0	Over	2	2	0
			Crustacean (e.g., *Daphnia magna*)			8.04	1.82	2.67 × 10	2	2	ND**	0	0	1
			Insect (e.g., *Aedes aegypti*)			7.02	4.96	7.28 × 10	1	3	Under	0	1	0
7	Thiophosphate	1/2/1	Fish (e.g., *Pimephales promelas*)	Malathion	Methyl parathion	7.14 × 10^3^	8.41 × 10^2^	1.31 × 10^3^	2	0	Over	1	0	0
			Crustacean (e.g., *Americamysis bahia*)			6.92 × 10^−1^	9.08 × 10^−1^	-	0	1	Under	0	2	0
			Insect (e.g., *Culex pipiens ssp.*)			3.07	8.21	1.28 × 10	0	2	Under	0	1	0
8	Thiophosphate	3/2/1	Fish (e.g., *Channa punctata*)	Malathion	Diazinon	1.44 × 10^3^	4.25 × 10^2^	5.41 × 10^2^	2	0	Over	3	0	0
			Crustacean (e.g., *Daphnia magna*)			1.22	1.74	2.30	0	3	Under	0	2	0
			Insect (e.g., *Culex quinquefasciatus*)			6.67	9.10	1.53 × 10	0	6	Under	0	1	0
9	Thiophosphate	1/1/10	Fish (e.g., *Pimephales promelas*)	Malathion	Fenthion	2.75 × 10^3^	2.28 × 10^2^	2.43 × 10^3^	10	0	Over	1	0	0
			Crustacean (e.g., *Americamysis bahia*)			2.65 × 10^−1^	1.58 × 10^−1^	1.69	1	9	Under	0	1	0
			Insect (e.g., *Aedes aegypti*)			2.35 × 10	1.04 × 10	-	1	0	Over	5	5	0
10	Thiophosphate	7/2/14	Fish (e.g., *Lepomis macrochirus*)	Malathion	Chlorpyrifos	5.72	2.86 × 10^−1^	9.82	22	6	Over	6	1	0
			Crustacean (e.g., *Americamysis bahia*)			4.00 × 10^−2^	1.90 × 10^−2^	1.42	5	93	Under	0	2	0
			Insect (e.g., *Aedes aegypti*)			7.02	1.05	4.29	14	0	Over	7	7	0
11	Thiophosphate	3/4/1	Fish (e.g., *Lepomis macrochirus*)	Methyl parathion	Chlorpyrifos	5.72	3.22 × 10^2^	1.38 × 10^3^	0	4	Under	0	3	0
			Crustacean (e.g., *Americamysis bahia*)			4.00 × 10^−2^	1.65 × 10^−2^	6.46 × 10^−2^	2	1	Over	3	1	0
			Insect (e.g., *Culex pipiens ssp.*)			1.04	2.05 × 10^−2^	3.45 × 10^−1^	12	0	Over	1	0	0
12	Thiophosphate	3/1/1	Fish (e.g., *Oncorhynchus mykiss*)	Diazinon	Fenthion	7.95 × 10^2^	5.88 × 10	-	1	0	Over	3	0	0
			Crustacean (e.g., *Americamysis bahia*)			2.65 × 10^−1^	9.99 × 10^−1^	1.19	0	3	Under	0	1	0
			Insect (e.g., *Culex pipiens ssp.*)			5.07	1.29 × 10	1.53 × 10	0	3	Under	0	1	0
13	Thiophosphate	6/3/2	Fish (e.g., *Lepomis macrochirus*)	Diazinon	Chlorpyrifos	5.72	1.80	8.05 × 10	1	5	Under	1	5	0
			Crustacean (e.g., *Americamysis bahia*)			4.00 × 10^−2^	4.61 × 10^−2^	1.26	0	12	Under	2	1	0
			Insect (e.g., *Culex pipiens ssp.*)			1.04	5.13 × 10^−1^	2.23 × 10	3	15	Under	1	1	0
14	Thiophosphate	5/1/11	Fish (e.g., *Oncorhynchus mykiss*)	Fenthion	Chlorpyrifos	1.82 × 10	5.86 × 10	1.77 × 10^2^	0	11	Under	0	5	0
			Crustacean (e.g., *Americamysis bahia*)			4.00 × 10^−2^	2.67 × 10^−3^	4.73 × 10^−2^	52	3	Over	1	0	0
			Insect (e.g., *Aedes aegypti*)			7.02	4.86 × 10^−1^	2.84	5	0	Over	9	2	0

**Table 4:** (Case study II) The example of results of the predicted values of acute aquatic toxicity (in read-across) for target chemicals using known toxicity data of source chemicals. * n: Number of species in fish/crustacean/insect.

No	Chemical Functional Group	n *	Species	Source Chemical	Target Chemical	Known Toxicity of Target Chemical (ug/L)	Predicted Toxicity of Target Chemical (Read-Across) (ug/L)	Known/ Predicted Toxicity Decision Ratio of Example	Known/Predicted Toxicity Decision Ratio of Each Species	
									Overestimation (n)	Underestimation (n)
1	Esters (phosphate)	3/2/0	Fish (e.g., *Lepomis macrochirus*)	Acephate	Methamidophos	4.13 × 10^4^	1.31 × 10^5^	Under	1	2
			Crustacean (e.g., *Americamysis bahia*)			1.05 × 10^3^	1.01 × 10^3^	Over	1	1
2	Esters (phosphate)	4/1/0	Fish (e.g., *Oncorhynchus mykiss*)	Acephate	Mevinphos	2.23 × 10	1.51 × 10^2^	Under	0	4
			Crustacean (e.g., *Americamysis bahia*)			1.30	1.92 × 10^−1^	Over	1	0
3	Esters (phosphate)	1/2/0	Fish (e.g., *Gambusia affinis*)	Acephate	Dichlorvos	5.27 × 10^3^	9.72 × 10^2^	Over	1	0
			Crustacean (e.g., *Americamysis bahia*)			2.26 × 10	1.23 × 10^2^	Under	0	2
4	Esters (phosphate)	2/1/0	Fish (e.g., *Oncorhynchus mykiss*)	Acephate	Nemacur	1.40 × 10^2^	7.91 × 10^2^	Under	0	2
			Crustacean (e.g., *Americamysis bahia*)			6.80	1.21	Over	1	0
5	Esters (phosphate)	1/3/1	Fish (e.g., *Gambusia affinis*)	Acephate	Profenofos	3.48 × 10^2^	1.02 × 10^2^	Over	1	0
			Crustacean (e.g., *Penaeus duorarum*)			4.60	3.95 × 10^2^	Under	1	2
			Insect (e.g, *Culex quinquefasciatus*)			1.18 × 10	1.38 × 10^−1^	Over	1	0
6	Esters (phosphate)	0/8/1	Crustacean (e.g., *Daphnia magna*)	Methamidophos	Dichlorvos	3.71	2.92 × 10	Under	0	8
			Insect (e.g, *Pteronarcys californica*)			2.50 × 10	1.79	Over	1	0
7	Esters (phosphate)	2/4/3	Fish (e.g., *Gambusia affinis*)	Dichlorvos	Profenofos	3.48 × 10^2^	2.00 × 10	Over	2	0
			Crustacean (e.g, *Ceriodaphnia dubia*)			4.10 × 10^−2^	1.26 × 10^−1^	Under	1	3
			Insect (e.g, *Culex pipiens*)			6.23 × 10	4.88	Over	2	1
8	Carbamate	2/1/0	Fish (e.g., *Pimephales promelas*)	Methomyl	Aminocarb	7.39 × 10^2^	3.72 × 10^2^	Over	1	1
			Crustacean (e.g, *Gammarus pseudolimnaeus*)			1.63 × 10^2^	2.42 × 10^2^	Under	0	1
9	Carbamate	1/2/0	Fish (e.g., *Oncorhynchus mykiss*)	Methomyl	Carbaryl	1.21 × 10^3^	2.11 × 10	Over	1	0
			Crustacean (e.g., *Americamysis bahia*)			1.01 × 10	5.67 × 10	Under	0	2
10	Carbamate	4/1/1	Fish (e.g., *Cyprinus carpio*)	Propoxur	Carbaryl	2.95 × 10^3^	3.27 × 10^3^	Under	2	2
			Crustacean (e.g., *Daphnia magna*)			2.07 × 10^2^	1.94 × 10^2^	Over	1	0
			Insect (e.g., *Aedes aegypti*)			7.08 × 10^2^	7.58 × 10^2^	Under	0	1
11	Carbamate	4/1/1	Fish (e.g., *Salvelinus fontinalis*)	Aminocarb	Carbaryl	1.88 × 10^3^	2.64 × 10^2^	Under	4	0
			Crustacean (e.g., *Gammarus pseudolimnaeus*)			1.53 × 10	1.11 × 10^2^	Over	0	1
			Insect (e.g., *Pteronarcella badia*)			1.53 × 10	2.12	Under	1	0
12	Thiophosphate	3/2/1	Fish (e.g., *Cyprinus carpio*)	Dimethoate	Malathion	2.65 × 10^3^	1.39 × 10^2^	Over	2	1
			Crustacean (e.g., *Daphnia magna*)			7.28	4.23 × 10	Under	1	1
			Insect (e.g., *Aedes aegypti*)			7.89 × 10	2.00 × 10^3^	Under	0	1
13	Thiophosphate	1/1/0	Fish (e.g., *Oncorhynchus mykiss*)	Dimethoate	Aldicarb	5.83 × 10^2^	1.32 × 10^3^	Under	0	1
			Crustacean (e.g., *Daphnia magna*)			3.19 × 10^2^	1.40 × 10^2^	Over	1	0
14	Thiophosphate	0/2/1	Crustacean (e.g., *Daphnia magna*)	Dimethoate	Dichlorvos	3.71	2.41 × 10	Under	1	1
			Insect (e.g., *Aedes aegypti*)			4.50 × 10	1.21 × 10^3^	Under	0	1
15	Thiophosphate	2/1/1	Fish (e.g., *Heteropneustes fossilis*)	Dimethoate	Propoxur	6.48 × 10^3^	3.80 × 10^3^	Over	1	1
			Crustacean (e.g., *Daphnia magna*)			3.76 × 10^2^	7.37 × 10^2^	Under	0	1
			Insect (e.g., *Aedes aegypti*)			1.37 × 10^3^	7.00 × 10^2^	Over	1	0
16	Thiophosphate	3/2/1	Fish (e.g., *Cyprinus carpio*)	Dimethoate	Carbaryl	2.95 × 10^3^	1.24 × 10^3^	Over	1	2
			Crustacean (e.g., *Daphnia magna*)			2.07 × 10^2^	3.80 × 10^2^	Under	0	2
			Insect (e.g., *Aedes aegypti*)			7.08 × 10^2^	3.40 × 10	Over	1	0
17	Thiophosphate	2/2/0	Fish (e.g., *Channa orientalis*)	Dimethoate	Methyl parathion	3.32 × 10^3^	2.05 × 10^2^	Over	2	0
			Crustacean (e.g., *Daphnia magna*)			1.65 × 10	1.28 × 10^3^	Under	0	2
18	Thiophosphate	3/1/0	Fish (e.g., *Cyprinus carpio*)	Dimethoate	Diazinon	8.72 × 10^2^	4.01	Over	3	0
			Crustacean (e.g., *Daphnia magna*)			1.22	2.67 × 10^2^	Under	0	1
19	Thiophosphate	1/0/1	Fish (e.g., *Oncorhynchus mykiss*)	Dimethoate	Fenthion	7.95 × 10^2^	5.25 × 10	Over	1	0
			Insect (e.g., *Aedes aegypti*)			2.35 × 10	3.56 × 10^2^	Under	0	1
20	Thiophosphate	4/1/1	Fish (e.g., *Oreochromis niloticus*)	Dimethoate	Chlorpyrifos	1.25 × 10^2^	5.30	Over	3	1
			Crustacean (e.g., *Daphnia magna*)			8.04	1.90 × 10^2^	Under	0	1
			Insect (e.g., *Aedes aegypti*)			7.02	1.50 × 10	Under	0	1
21	Thiophosphate	8/3/0	Fish (e.g., *Cyprinodon variegatus*)	Malathion	Azinphos-methyl	2.28	2.98	Under	5	3
			Crustacean (e.g., *Gammarus fasciatus*)			2.01 × 10^−1^	3.60 × 10^−2^	Over	3	0
22	Thiophosphate	1/2/1	Fish (e.g., *Pimephales promelas*)	Malathion	Methyl parathion	7.14 × 10^3^	7.93 × 10^2^	Over	1	0
			Crustacean (e.g., *Americamysis bahia*)			6.92 × 10^−1^	3.21 × 10^−1^	Over	1	1
			Insect (e.g., *Culex pipiens ssp. Quinquefasciata*)			3.07	6.63	Under	0	1
23	Thiophosphate	3/2/1	Fish (e.g., *Channa punctata*)	Malathion	Diazinon	1.44 × 10^3^	4.31 × 10^2^	Over	3	0
			Crustacean (e.g., *Ceriodaphnia dubia*)			4.23 × 10^−1^	2.68 × 10^−1^	Over	1	1
			Insect (e.g., *Culex quinquefasciatus*)			6.67	6.50	Over	1	0
24	Thiophosphate	1/1/10	Fish (e.g., *Pimephales promelas*)	Malathion	Fenthion	2.75 × 10^3^	7.03 × 10	Over	1	0
			Crustacean (e.g, *Americamysis bahia*)			2.65 × 10^−1^	5.29 × 10^−1^	Under	0	1
			Insect (e.g., *Culex pipiens ssp. Quinquefasciata*)			5.07	2.54	Over	7	3
25	Thiophosphate	7/3/16	Fish (e.g., *Lepomis macrochirus*)	Malathion	Chlorpyrifos	5.72	6.70	Under	6	1
			Crustacean (e.g., *Ceriodaphnia dubia*)			6.74 × 10^−2^	8.65 × 10^−1^	Under	0	3
			Insect (e.g., *Chironomus utahensis*)			1.98	3.66 × 10^−2^	Over	9	7
26	Thiophosphate	2/2/0	Fish (e.g., *Lepomis macrochirus*)	Azinphos-methyl	Chlorpyrifos	5.72	4.90	Over	1	1
			Crustacean (e.g., *Americamysis bahia*)			4.00 × 10^−2^	1.07 × 10^−1^	Under	0	2
27	Thiophosphate	3/2/0	Fish (e.g., *Lepomis macrochirus*)	Methyl parathion	Diazinon	2.00 × 10^2^	2.53 × 10^2^	Under	2	1
			Crustacean (e.g., *Ceriodaphnia dubia*)			4.23 × 10^−1^	1.49 × 10^−1^	Over	2	0
28	Thiophosphate	4/1/0	Fish (e.g., *Morone saxatilis*)	Methyl parathion	Fenthion	4.53 × 10^2^	8.89 × 10^2^	Under	2	2
			Crustacean (e.g., *Americamysis bahia*)			2.65 × 10^−1^	1.35 × 10^−1^	Over	1	0
29	Thiophosphate	3/4/1	Fish (e.g., *Lepomis macrochirus*)	Methyl parathion	Chlorpyrifos	5.72	3.74 × 10^2^	Under	0	3
			Crustacean (e.g., *Americamysis bahia*)			4.00 × 10^−2^	2.33 × 10^−1^	Under	2	2
			Insect (e.g., *Culex pipiens ssp. Quinquefasciata*)			1.04	8.15 × 10^−2^	Over	1	0
30	Thiophosphate	3/1/1	Fish (e.g., *Oncorhynchus mykiss*)	Diazinon	Fenthion	7.95 × 10^2^	6.70 × 10	Over	3	0
			Crustacean (e.g., *Americamysis bahia*)			2.65 × 10^−1^	3.44 × 10^−1^	Under	0	1
			Insect (e.g., *Culex pipiens ssp. Quinquefasciata*)			5.07	3.91	Over	1	0
31	Thiophosphate	3/1/0	Fish (e.g., *Lepomis macrochirus*)	Diazinon	EPN	1.36 × 10^2^	1.57 × 10^2^	Under	0	3
			Crustacean (e.g., *Americamysis bahia*)			4.63	4.00	Over	1	0
32	Thiophosphate	6/3/2	Fish (e.g., *Lepomis macrochirus*)	Diazinon	Chlorpyrifos	5.72	2.38	Over	4	2
			Crustacean (e.g., *Ceriodaphnia dubia*)			5.69 × 10^−2^	1.30 × 10^−1^	Under	1	2
			Insect (e.g., *Culex quinquefasciatus*)			2.25	4.54 × 10^−2^	Over	2	0
33	Thiophosphate	2/1/0	Fish (e.g., *Pimephales promelas*)	Disulfoton	EPN	8.81 × 10	8.30 × 10^2^	Under	0	2
			Crustacean (e.g., *Gammarus fasciatus*)			6.87	7.29 × 10^−1^	Over	1	0
34	Thiophosphate	5/1/11	Fish (e.g., *Oncorhynchus mykiss*)	Fenthion	Chlorpyrifos	1.82 × 10	2.86 × 10	Under	2	3
			Crustacean (e.g., *Americamysis bahia*)			4.00 × 10^−2^	8.19 × 10^−2^	Under	0	1
			Insect (e.g., *Culex tarsalis*)			5.30 × 10^−1^	2.59 × 10^−1^	Over	5	6
35	Thiophosphate	2/1/0	Fish (e.g., *Lepomis macrochirus*)	EPN	Chlorpyrifos	5.72	1.17	Over	2	0
			Crustacean (e.g., *Americamysis bahia*)			4.00 × 10^−2^	1.95 × 10^−1^	Under	0	1

**Table 5:** Lack of agreement (bias, relative bias, precision, and accuracy) for a new read-across concept approach in Case study I and II.

Chemical Functional Group	n	New Read-Across Concept			
		Bias	Relative Bias (%)	Precision	Accuracy
Case study I					
Overall	1095	0.32	37.65	0.01	0.010
Esters (phosphate)	81	0.15	15.70	0.05	0.050
Carbamate	24	0.16	17.51	0.01	0.050
Thiophosphate	990	0.01	1.24	0.01	0.004
Case study II					
Overall	218	0.65	91.94	0.06	0.050
Esters (phosphate)	39	0.36	43.18	0.15	0.150
Carbamate	18	0.23	25.26	0.03	0.080
Thiophosphate	161	0.07	7.02	0.02	0.020

## Data Availability

The data will be made available upon request by means of a project agreement from the authors. Requests should be sent to haha0694@skuniv.ac.kr or jinsungra@kitech.re.kr and are subject to approval by all named authors participating in this study.

## Supplementary Materials

The following supporting information can be downloaded at: https://www.mdpi.com/article/10.3390/toxics12010096/s1, Table S1: Physicochemical properties and classification of aquatic toxicity for 25 target chemical substances included in this study. Table S2: Acute aquatic toxicity data collected from the ECOTOXicology Knowledgebase (ECOTOX, Version 5, US EPA, 2022) for 25 target chemicals.
