# Supplementary material for: Developing a Novel Read-Across Concept for Ecotoxicological Risk Assessment of Phosphate Chemicals: A Case Study
Source: Toxics. 2024 Jan 22;12(1):96. doi: 10.3390/toxics12010096 (PMC10818528; doi:10.3390/toxics12010096)
Supplement: Supplementary file 1 [file toxics-12-00096-s001.zip › toxics-2787189-supplementary.pdf]

Supplementary Table S1. Physicochemical properties and classification of aquatic toxicity for 25 target chemical substances included in this study. [All pictures of chemical structures were downloaded from an official website of PubChem (https://pubchem.ncbi.nlm.nih.gov/).].

No	Chemical name	CAS no.	Molecular formula	Chemical structure	EU CLP harmonized classification	Hazard statement code
1	Acephate	30560-19-1	C4H10NO3PS	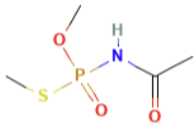	Not classified	Not classified
2	Dichlorvos	62-73-7	C4H7Cl2O4P	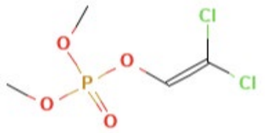	Aquatic Actue 1	H400
3	Methamidophos	10265-92-6	C2H8NO2PS	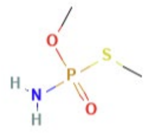	Aquatic Actue 1	H400
4	Mevinphos	7786-34-7	C7H13O6P	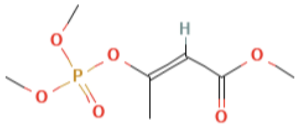	Aquatic Actue 1<br>Aquatic Chronic 1	H400<br>H410
5	Nemacur	22224-92-6	C13H22NO3PS	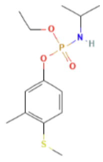	Aquatic Actue 1<br>Aquatic Chronic 1	H400<br>H410
6	Profenofos	41198-08-7	C11H15BrClO3PS	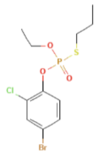	Aquatic Actue 1<br>Aquatic Chronic 1	H400<br>H410
7	Aminocarb	2032-59-9	C11H16N2O2	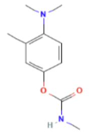	Aquatic Actue 1<br>Aquatic Chronic 1	H400<br>H410
8	Carbaryl	63-25-2	C12H11NO2	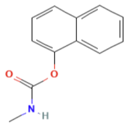	Aquatic Actue 1	H400
9	Carbofuran	1563-66-2	C12H15NO3	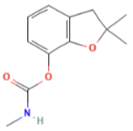	Aquatic Actue 1<br>Aquatic Chronic 1	H400<br>H410
10	Propoxur	114-26-1	C11H15NO3	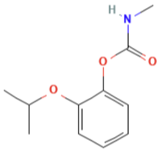	Aquatic Actue 1<br>Aquatic Chronic 1	H400<br>H410
11	Aldicarb	116-06-3	C7H14N2O2S	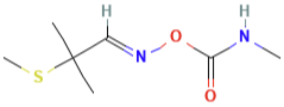	Aquatic Actue 1<br>Aquatic Chronic 1	H400<br>H410
12	Methomyl	16752-77-5	C5H10N2O2S	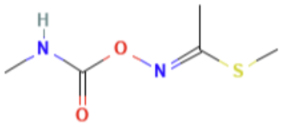	Aquatic Actue 1<br>Aquatic Chronic 1	H400<br>H410

13	Oxamyl	23135-22-0	C7H13N3O3S	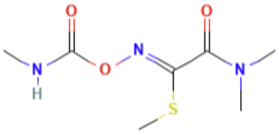	Aquatic Chronic 2	H411
14	Chlorpyrifos	2921-88-2	C9H11Cl3NO3PS	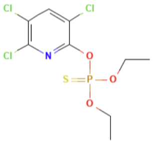	Aquatic Actue 1<br>Aquatic Chronic 1	H400<br>H410
15	Diazinon	333-41-5	C12H21N2O3PS	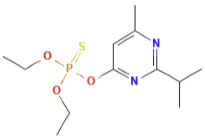	Aquatic Actue 1<br>Aquatic Chronic 1	H400<br>H410
16	EPN	2104-64-5	C14H14NO4PS	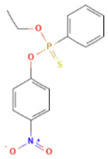	Aquatic Actue 1<br>Aquatic Chronic 1	H400<br>H410
17	Fensulfothion	115-90-2	C11H17O4PS2	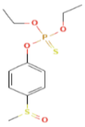	Aquatic Actue 1<br>Aquatic Chronic 1	H400<br>H410
18	Fenthion	55-38-9	C10H15O3PS2	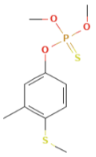	Aquatic Actue 1<br>Aquatic Chronic 1	H400<br>H410
19	Methyl parathion	298-00-0	C8H10NO5PS	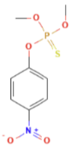	Aquatic Actue 1<br>Aquatic Chronic 1	H400<br>H410
20	Azinphos-methyl	86-50-0	C10H12N3O3PS2	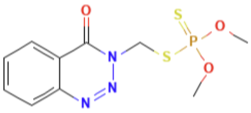	Aquatic Actue 1<br>Aquatic Chronic 1	H400<br>H410
21	Dimethoate	60-51-5	C5H12NO3PS2	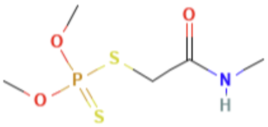	Not classified	Not classified
22	Disulfoton	298-04-4	C8H19O2PS3	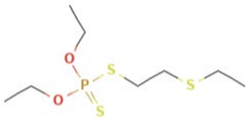	Aquatic Actue 1<br>Aquatic Chronic 1	H400<br>H410
23	Dyfonate	944-22-9	C10H15OPS2	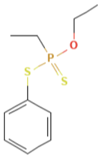	Aquatic Actue 1<br>Aquatic Chronic 1	H400<br>H410
24	Malathion	121-75-5	C10H19O6PS2	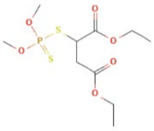	Aquatic Actue 1<br>Aquatic Chronic 1	H400<br>H410
25	Terbufos	13071-79-9	C9H21O2PS3	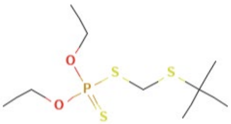	Aquatic Actue 1<br>Aquatic Chronic 1	H400<br>H410

Supplementary Table S2. Acute aquatic toxicity data collected from the ECOTOXicology Knowledgebase (ECOTOX, Version 5, US EPA, 2022) for 25 target chemicals. * LC_{50}(mg/L) : Any of available toxicity values (either 24hr, 48hr, or 96hr-LC_{50}) was selected and collected from the ECOTOX database.

No	Chemical functional group	Chemical name	Fish		Crustacean		Insect		Amphibian	
			n	Species	n	Species	n	Species	n	Species
1	Esters (phosphate)	Acephate	12	Oncorhynchus mykiss, Mystus vittatus, Lepomis macrochirus, Clarias batrachus, etc.	7	Litopenaeus vannamei, Homarus americanus, Americamysis bahia, Penaeus aztecus, etc.	9	Aedes aegypti, Culex quinquefasciatus, Isogenus sp., Pteronarcella badia, etc.	2	Ambystoma gracile, Lithobates clamitans sp.
2		Dichlorvos	38	Danio rerio, Cyprinodon variegatus, Abramis brama, Heteropneustes fossilis, etc.	25	Gammarus lacustris, Gammarus fasciatus, Daphnia magna, Pagurus longicarpus, Scylla serrata, etc.	24	Peltodytes sp., Culex pipiens ssp. Pallens, Aedes aegypti, Pteronarcys californica, etc.	5	Duttaphrynus melanostictus, Fejervarya multistriata, Microhyla ornata, Polypedates megacephalus, etc.
3		Methamidophos	8	Cyprinodon variegatus, Lepomis macrochirus, Cyprinus carpio, etc.	4	Macrobrachium rosenbergii, Daphnia magna, etc.	-	-	1	Rana limnocharis
4		Mevinphos	12	Oncorhynchus mykiss, Anguilla rostrata, Mugil cephalus, Carassius auratus, etc.	9	Pagurus longicarpus, Ceriodaphnia dubia, Crangon septemspinosa, Gammarus fasciatus, etc.	1	Pteronarcys californica	-	-
5		Nemacur	3	Cyprinodon variegatus, Lepomis macrochirus, Oncorhynchus mykiss, etc.	6	Daphnia carinata, Americamysis bahia, Daphnia pulex, Gammarus italicus, etc.	-	-	-	-
6		Profenofos	17	Channa punctata, Ameiurus melas, Cyprinus carpio, Oncorhynchus mykiss, etc.	11	Artemia salina, Macrobrachium rosenbergii, Caridina laevis, Ceriodaphnia dubia, etc.	6	Aedes albopictus, Chironomus tentans, Culex pipiens, etc.	1	Quasipaa spinosa
7	Carbamate	Aminocarb	12	Oncorhynchus mykiss, Salmo salar, Salvelinus fontinalis, Perca flavescens, etc.	8	Gammarus fasciatus, Gammarus pseudolimnaeus, Penaeus aztecus, Orconectes sp., etc.	7	Acroneuria sp., Pteronarcella badia, Skwala sp., Isonychia sp., etc.	-	-
8		Carbaryl	60	Morone saxatilis, Oncorhynchus clarkii ssp. Stomias, Cyprinus carpio, Leuciscus cephalus, etc.	41	Paratelphusa masoniana, Gammarus pulex, Daphnia magna, Orconectes nais, etc.	42	Aedes aegypti, Chauliodes sp., Pteronarcys californica, Brachythemis contaminata, etc.	17	Lithobates clamitans ssp. Clamitans, Xenopus laevis, Pseudacris regilla, etc.
9		Carbofuran	28	Morone saxatilis, Lepomis macrochirus, Cyprinus carpio, Oryzias latipes, Salvelinus namaycush, etc.	15	Caridina rajadhari, Paratelphusa jacquemontii, Ceriodaphnia dubia, Caridina laevis, etc.	8	Anisops bouvieri, Brachythemis contaminata, Chironomus riparius, Tanypus bilobatus, etc.	3	Euphlyctis hexadactylus(, Microhyla ornata, Rana limnocharis, Rana boylii
10		Propoxur	12	Cyprinus carpio, Oncorhynchus mykiss, Tinca tinca, etc.	7	Americamysis bahia, Gammarus italicus, Daphnia magna, etc.	22	Aedes aegypti, Culex quinquefasciatus, Lestes congener, Peltodytes sp., etc.	-	-
11		Aldicarb	9	Oryzias latipes, Lagodon rhomboides, Oncorhynchus mykiss, etc.	16	Daphnia magna, Daphnia laevis, Hyalella azteca, Artemia sp., etc.	5	Aedes aegypti, Aedes taeniorhynchus, Chironomus riparius, Culex quinquefasciatus	-	-
12		Methomyl	14	Menidia menidia, Oncorhynchus mykiss, Salmo salar, etc.	19	Daphnia magna, Penaeus duorarum, Artemia salina, Gammarus pulex, etc.	4	Aedes albopictus, Culex pipiens, Isogenus sp., Pteronarcella badia	3	Duttaphrynus melanostictus, Microhyla pulchra, Polypedates megacephalus

13		Oxamyl	6	Cyprinodon variegatus, Oncorhynchus mykiss, Pimephales promelas, etc.	4	Gammarus italicus, Echinogammarus tibaldii, Palaemonetes pugio,	-	-	-	-
14	Thiophosphate	Chlorpyrifos	40	Morone saxatilis, Danio rerio, Oncorhynchus mykiss, Pungitius pungitius, etc.	47	Artemia sp., Daphnia pulex, Daphnia magna, Procambarus clarkii, Neocaridina denticulata, etc.	85	Culex pipiens, Parapoynx stratiotata, Pteronarcys californica, Notonecta maculata, etc.	6	Hoplobatrachus tigerinus, Bufo bufo ssp. Gargarizans, Xenopus laevis, etc.
15		Diazinon	53	Anguilla anguilla, Oncorhynchus mykiss, Danio rerio, Barbus grypus, etc.	20	Ceriodaphnia dubia, Daphnia magna, Americamysis bahia, Palaemon adspersus, etc.	23	Aedes cantans, Attaneuria ruralis, Chironomus tentans, Procloeon sp., etc.	5	Pseudacris regilla, Rana boylii, Rana limnocharis, Rhinella arenarum, Xenopus laevis
16		EPN	11	Morone saxatilis, Oncorhynchus mykiss, Micropterus salmoides, etc.	6	Palaemonetes kadiakensis, Gammarus fasciatus, Penaeus stylirostris, etc.	2	Culex tritaeniorhynchus, Pteronarcys californica	-	-
17		Fensulfothion	3	Mystus cavasius, Lepomis macrochirus, Pimephales promelas	2	Gammarus fasciatus, Daphnia magna	-	-	-	-
18		Fenthion	21	Morone saxatilis, Oncorhynchus mykiss, Cyprinus carpio, Tilapia rendalli, etc.	16	Penaeus duorarum, Americamysis bahia, Daphnia magna, etc.	57	Aedes aegypti, Chauliodes sp., Chironomus yoshimatsui, Pteronarcys californica, etc.	1	Euphlyctis hexadactylus
19		Methyl parathion	42	Morone saxatilis, Oncorhynchus mykiss, Cyprinus carpio, Oryzias latipes, etc.	28	Daphnia magna, Penaeus duorarum, Oziotelphusa senex ssp. Senex, etc.	12	Culex tarsalis, Ischnura verticalis, Procladius sp., Chironomus sp., etc.	4	Hoplobatrachus tigerinus, Pseudacris triseriata, Rana cyanophlyctis, etc.
20		Azinphos-methyl	26	Cyprinus carpio, Oncorhynchus mykiss, Ameiurus melas, etc.	10	Americamysis bahia, Palaemonetes pugio, Daphnia magna, etc.	6	Chironomus sp., Chironomus tentans, Pteronarcys californica, etc.	-	-
21		Dimethoate	26	Danio rerio, Oncorhynchus mykiss, Clarias gariepinus, Barbus ticto, etc.	14	Gammarus pulex, Gammarus lacustris, Daphnia magna, Artemia sp., etc.	13	Aedes aegypti, Chironomus dilutus, Pteronarcys californica, Kiefferulus sp., etc.	5	Euphlyctis hexadactylus, Hyla versicolor, Polypedates cruciger, Rana cyanophlyctis, Rana limnocharis
22		Disulfoton	10	Anguilla anguilla, Oncorhynchus mykiss, Carassius auratus, etc.	3	Americamysis bahia, Gammarus fasciatus, Palaemonetes kadiakensis	1	Pteronarcys californica	-	-
23		Dyfonate	5	Cyprinus carpio, Cyprinodon variegatus, Lepomis macrochirus, Oncorhynchus mykiss	2	Americamysis bahia, Daphnia magna	2	Culex pipiens ssp. Quinquefasciata, Chironomus riparius	-	-
24		Malathion	72	Barbus stigma, Oncorhynchus mykiss, Perca flavescens, etc.	46	Gammarus pulex, Gammarus lacustris, Daphnia magna, Artemia salina, etc.	67	Aedes aegypti, Anisops sardeus, Hydrophilus sp., Simulium vittatum, etc.	9	Euphlyctis cyanophlyctis, Pseudacris triseriata, Rana limnocharis, etc.
25		Terbufos	8	Atherinops affinis, Oncorhynchus mykiss, Salmo trutta, etc.	5	Americamysis bahia, Palaemonetes sp., Procambarus clarkii, etc.	-	-	-	-
